# *Restorer-of-Fertility* Mutations Recovered in Transposon-Active Lines of S Male-Sterile Maize

**DOI:** 10.1534/g3.117.300304

**Published:** 2017-11-22

**Authors:** Susan Gabay-Laughnan, A. Mark Settles, L. Curtis Hannah, Timothy G. Porch, Philip W. Becraft, Donald R. McCarty, Karen E. Koch, Liming Zhao, Terry L. Kamps, Karen C. Chamusco, Christine D. Chase

**Affiliations:** * Department of Plant Biology, University of Illinois, Urbana, Illinois 61801; † Horticultural Sciences Department, University of Florida, Gainesville, Florida 32611; ‡ Tropical Agriculture Research Station, The United States Department of Agriculture, Agriculture Research Service, Mayaguez, Puerto Rico 00680-5470; § Department of Genetics, Development, and Cell Biology, Iowa State University, Ames, Iowa 50011; ** Department of Agronomy, Iowa State University, Ames, Iowa 50011; †† Florida Medical Entomology Laboratory, Vero Beach, Florida 32962; ‡‡ Biology Department, New Jersey City University, Jersey City, NJ 07305

**Keywords:** cytoplasmic male sterility, pollen, seed, gametophyte, mitochondria

## Abstract

Mitochondria execute key pathways of central metabolism and serve as cellular sensing and signaling entities, functions that depend upon interactions between mitochondrial and nuclear genetic systems. This is exemplified in cytoplasmic male sterility type S (CMS-S) of *Zea mays*, where novel mitochondrial open reading frames are associated with a pollen collapse phenotype, but nuclear *restorer-of-fertility* (restorer) mutations rescue pollen function. To better understand these genetic interactions, we screened *Activator-Dissociation* (*Ac-Ds*), *Enhancer/Suppressor-mutator* (*En/Spm*), and *Mutator* (*Mu*) transposon-active CMS-S stocks to recover new restorer mutants. The frequency of restorer mutations increased in transposon-active stocks compared to transposon-inactive stocks, but most mutants recovered from *Ac-Ds* and *En/Spm* stocks were unstable, reverting upon backcrossing to CMS-S inbred lines. However, 10 independent restorer mutations recovered from CMS-S *Mu* transposon stocks were stable upon backcrossing. Many restorer mutations condition seed-lethal phenotypes that provide a convenient test for allelism. Eight such mutants recovered in this study included one pair of allelic mutations that were also allelic to the previously described *rfl2-1* mutant. Targeted analysis of mitochondrial proteins by immunoblot identified two features that consistently distinguished restored CMS-S pollen from comparably staged, normal-cytoplasm, nonmutant pollen: increased abundance of nuclear-encoded alternative oxidase relative to mitochondria-encoded cytochrome oxidase and decreased abundance of mitochondria-encoded ATP synthase subunit 1 compared to nuclear-encoded ATP synthase subunit 2. CMS-S restorer mutants thus revealed a metabolic plasticity in maize pollen, and further study of these mutants will provide new insights into mitochondrial functions that are critical to pollen and seed development.

Mitochondria are the cellular site of the tricarboxylic acid cycle, respiratory electron transfer, and ATP synthesis, which are all critical pathways of central metabolism (Schertl and Braun 2014). These organelles are also an important source of biosynthetic intermediates and cellular signaling molecules (Jacoby *et al.* 2012; Schwarzländer and Finkemeier 2013). Mitochondrial processes depend upon the coordinated function of the mitochondrial and nuclear genomes (Colas des Francs-Small and Small 2014), with over 2000 nuclear genes encoding proteins that are translated in the cytosol and imported into the mitochondria (Emanuelsson *et al.* 2000).

CMS systems establish a link between mitochondrial function and pollen development, and also exemplify interactions between nuclear and mitochondrial genetic systems. CMS is a mitochondria-encoded failure to produce or release functional pollen (Horn *et al.* 2014). This is a common type of mutation in the angiosperms (Laser and Lersten 1972), and male sterility can be manifested in a variety of phenotypes, including homeotic changes in floral organ identity, abortion of pollen producing organs, or abortion of the developing pollen itself (Carlsson *et al.* 2008; Linke and Börner 2006). These diverse phenotypes are conditioned by different CMS genes that are typically comprised of segments derived from mitochondrial gene coding and flanking sequences (Hanson and Bentolila 2004) spliced together by highly active plant mitochondrial genome recombination processes (Davila *et al.* 2011; Gualberto *et al.* 2014; Maréchal and Brisson 2010). CMS can be reversed or suppressed by system-specific nuclear restorer genes (Chase 2007; Chen and Liu 2014). Many restorers encode members of the pentatricopeptide repeat (PPR) protein family. This is a highly expanded gene family in plants, with over 400 members in *Arabidopsis* (Lurin *et al.* 2004). Almost all are targeted to plastids or mitochondria, where they function as site-specific RNA binding proteins that mediate key organelle gene expression processes of transcription, processing, splicing, editing, or translation (Barkan and Small 2014; Manna 2015). Restorers and restorer-like PPR proteins comprise a separate clade from other mitochondria-targeted PPRs. Moreover, genes encoding PPRs that fall into this clade are often found in clusters of duplicated genes that have undergone rapid evolution and diversifying selection. PPR-encoding restorer genes are therefore considered to be the result of adaptive evolution for the silencing of specific mitochondrial CMS genes (Dahan and Mireau 2013; Fujii *et al.* 2011; Gaborieau *et al.* 2016; Melonek *et al.* 2016).

CMS-S maize offers a different paradigm for fertility restoration compared to those described above. In this gametophytic system of CMS and fertility restoration, the molecular and cellular events that determine pollen fertility occur in the developing haploid male gametophyte. In S cytoplasm, pollen containing a nuclear restorer allele will function, whereas pollen without a restoring allele will collapse (Buchert 1961). While there are native restorers for S-cytoplasm maize, *Rf3* (Buchert 1961) and *Rf9* (Gabay-Laughnan *et al.* 2009), CMS-S maize is the only system where restorers are reported to arise in real time through genetic mutation (Gabay-Laughnan *et al.* 1995; Laughnan and Gabay 1973, 1978). These are observed as fertile tassel sectors or entirely fertile tassels on CMS-S maize plants, and are recovered by crosses with the pollen from these sectors. While each new restorer rescues CMS-S pollen function, many also condition a homozygous-lethal phenotype with respect to seed development (Laughnan and Gabay 1978). These *restorer-of-fertility lethal* (*rfl*) mutations include recessive, loss-of-function mutations (Wen *et al.* 2003) that are hypothesized to disrupt the expression of CMS-S in pollen at the expense of mitochondrial functions essential to seed development. Consistent with this hypothesis, the spontaneous *rfl1-1* allele cosegregates with loss of mitochondria-encoded ATP synthase subunit 1 (ATP1) (Wen *et al.* 2003). The *rfl* seed phenotypes provide a convenient means of testing allelism between independent *rfl* mutants. The collection of spontaneous *rfl* mutants currently includes 41 nonallelic mutants (S. Gabay-Laughnan, unpublished data). *Restorer-of-fertility viable* (*rfv*) mutations that do not condition seed lethality have also been recovered (Laughnan and Gabay 1978). The *rfl* and *rfv* mutants contrast to the native restorers, which have genetic features consistent with adaptive evolution for the silencing of CMS-S. *Rf3* is a dominant (gain-of-function) allele (Kamps *et al.* 1996) that is present in Mexican landraces and in the teosinte progenitor of modern maize (Gabay-Laughnan *et al.* 2004). Moreover, the *rf3* locus maps to a cluster of related PPR protein genes (Xu *et al.* 2009).

The loss-of-function nature of the *rfl* (and possibly *rfv*) mutants indicates that such mutants could be induced by transposon mutagenesis. Transposon-induced mutants would facilitate gene cloning and identification by transposon-tagging strategies (Brutnell 2002; Walbot 2000). The maize *Ac-Ds* (Lazarow *et al.* 2013), *En/Spm* (Cone *et al.* 1986; O’Reilly *et al.* 1985), and *Mu* (McCarty *et al.* 2013; Williams-Carrier *et al.* 2010) elements have all been used to generate novel mutants and to subsequently clone the tagged genes. Here, we exploited the features of CMS-S along with maize transposable element tools to generate and further characterize a collection of these unusual fertility restorer alleles.

## Materials and Methods

### Genetic materials and nomenclature

The maize stocks used in this work are summarized in Table 1. In maize genetic nomenclature (http://www.maizegdb.org/nomenclature, accessed 8 May, 2017), loci are indicated in lowercase italics (*e.g.*, the *rf3* locus). Alleles at a locus are also indicated in italics, with the first letter capitalized for dominant alleles (*e.g.*, the *Rf3* allele). We adopted the symbol *rfl* for homozygous-lethal restorers (Wen *et al.* 2003). Homozygous-viable restorers that arise via mutation are designated *rfv*, although the dominant or recessive nature of these alleles cannot be known for certain without functional tests performed in diploid pollen (Kamps *et al.* 1996). The symbol * and a laboratory number that indicates the year of isolation and the field row number identify restorer alleles that have yet to be mapped or tested for allelism with those already mapped. Plant mitochondrial genes are also indicated in lowercase italics (*e.g.*, the *atp1* gene). The corresponding protein product is indicated in uppercase without italics (*e.g.*, the ATP1 protein). The normal cytoplasm of maize, which does not induce pollen sterility, is designated N. The S male sterility-inducing cytoplasm is designated CMS-S or, briefly, S. Since there have been numerous independent recoveries of S-type cytoplasms, subgroups or subtypes of CMS-S have been given unique letter designations [Beckett 1971; Sisco *et al.* 1985; reviewed in Gabay-Laughnan *et al.* (1995)]. The subtypes of CMS-S used in this study were S, ML, R, and VG.

**Table 1:** Genetic materials

Stock	Cytoplasm	Nucleus*^a^*
N-H109	N*^b^*	H109
N-ccB73 *Mu*-on*^c^*	N	ccCB73 *Mu*-on
N-Mo17	N	Mo17
S-Mo17	CMS-S	Mo17
S-Mo17/B73	CMS-S	Mo17/B73*^d^*
S-Mo17 *Rf3-CE1*	CMS-S	*Rf3-CE1**^e^**/rf3-Mo17*
N-Oh545	N	Oh545
VG-W23	CMS-S*^f^*	W23
ML-W64A	CMS-S*^f^*	W64A
*a1-m1*	N	*a1-m1*::*I**^g^* *sh2 En*(*Spm*)*/a1 Sh2*_*^h^*
*a1-m4*	N	*a1-m4*::*Ds**^i^* *Sh2/a1-s Sh2*; *Ac/_**^j^*

N, normal cytoplasm; CMS-S, cytoplasmic male sterility type S.

a Nuclear genotypes do not carry restorers for CMS-S unless indicated.

b Normal cytoplasm that does not induce pollen sterility.

c Color-converted, transposon *Mu*-active derivative of the B73 inbred line.

d Mo17-B73 hybrid nucleus.

e *Rf3-CE1* was backcrossed to Mo17 for 17 generations.

f VG and ML are subgroups of CMS-S.

g Nonautonomous element transposes in response to the autonomous *En*(*Spm*).

h No *En*(*Spm*) present.

i Nonautonomous element transposes in response to the autonomous *Ac*.

j No *Ac* present.

### Mutant screens

Mutant screens of CMS-S *Ac-Ds* and CMS-S *En/Spm* stocks for new restorer mutants were conducted at the University of Illinois research farm in Urbana-Champaign, IL. For mutant screen 1, we developed Mo17 *Ac-Ds* transposon-active stocks in both N and CMS-S cytoplasms, as outlined in Supplemental Material, Figure S1 in File S1. CMS-S Mo17 exhibits a very low rate of spontaneous reversion to pollen fertility (Gabay-Laughnan and Chase 2000). We therefore expected a low rate of background, spontaneous restorer mutations in the Mo17-converted transposon stocks. We screened 1241 CMS-S *Ac-Ds*-carrying plants for new restorer mutants. For mutant screen 2, *En/Spm* stocks were developed in S and N cytoplasms, as outlined in Figure S2 in File S1. We searched 1433 CMS-S plants (an estimated 1239 carrying *En/Spm*) for restorer mutants. In both of these screens, CMS-S seeds carrying active transposons were not bulked but were instead planted one ear per row. This allowed for the identification of possible ear sectors, *i.e.*, multiple kernels carrying the same mutation. In our screens, the plants are male-sterile and the desired mutants are male-fertile. Plants were screened daily at maturity to identify plants with fully fertile tassels or those with sectors of pollen fertility. Plants carrying putative restoring alleles were crossed as pollen parents onto ears of CMS-S pollen-sterile testers to confirm and recover any pollen-transmissible nuclear mutations that restored male fertility in the presence of CMS-S.

Mutant screens of CMS-S *Mu* transposon-active stocks were conducted at the University of Florida Plant Science Research Unit located in Citra, FL. A color-converted B73 (ccB73) UniformMu resource was developed, originally for the recovery of seed development mutants, as outlined in Figure S3A in File S1. We subsequently investigated this resource as a source of *Mu* transposon-induced *rfl* mutants, as outlined in Figure S3B in File S1. The ccB73 version of UniformMu was selected for this screen because the B73 inbred line is known to maintain CMS-S pollen sterility. To confirm maintenance of pollen sterility in the ccB73 UniformMu background, CMS-S Mo17 was pollinated with ccB73 UniformMu, *Mu*-active plants grown from two ears that were not segregating seed mutants. Thirty progeny from each of the two resulting families were grown and examined for pollen fertility. The largely pollen-sterile families confirmed that fertility restorers were not uniformly present in the ccB73 UniformMu background. We subsequently grew plump kernels from 22 N-cytoplasm ccB73 UniformMu, *Mu*-inactive ears segregating for independent, seed-lethal mutants and crossed the resulting plants with pollen-sterile CMS-S Mo17/B73 hybrid plants. In mutant screen 3, 22 progeny families of 15 plants each (one family tracing back to each of the 22 ccB73 UniformMu ears) were grown and screened for pollen fertility. Pollen-shedding plants were backcrossed as pollen parents onto ears of CMS-S Mo17/B73 hybrid plants to confirm and recover any pollen-transmissible nuclear mutations that restored pollen fertility in the presence of CMS-S.

### Mutant classification

CMS-S plants heterozygous for new restorer mutants were self- or sib-pollinated to test for homozygous viability *vs.* homozygous lethality of the new restorer allele. Restorers were assigned to the *rfl* class if the resulting ears segregated ∼1:1 for aborted and normal seeds counted on the ear. The 1:1 segregation is diagnostic because, in S cytoplasm, only the pollen carrying a restoring allele (here an *rfl* mutant) will function. Pollen carrying the nonrestoring allele is collapsed (Buchert 1961). New restorers were designated *rfv* if self- or sib-ears had full, normal seed sets. In a few cases, classifications were in question because of poor seed sets (possibly due to *rfl* mutants conditioning semisterile seed set lethality) or the segregation of < 50% aborted kernels (possibly due to a seed-lethal mutant loosely linked to a restorer mutant). In each of these cases, the *rfv* assignment was ruled out based upon the absence of plants homozygous for fertility restoration when seeds from self- or sib-progeny ears were grown. Homozygosity and heterozygosity were determined based upon microscopic examination of pollen, as described by Gabay Laughnan *et al.* (2009) or by Kamps *et al.* (1996). Homozygous restored plants have pollen fertility approaching 100%, whereas heterozygous restored plants produce 50% collapsed pollen. Given CMS-S parent plants heterozygous for an *rfv* allele (*i.e.*, *rfv/Rfv*), self- or sib-progenies are expected to segregate 1:1 for *rfv/Rfv* heterozygous:*rfv*/*rfv* homozygous restored genotypes.

### Tests of allelism

The seed-lethal trait was used as the basis for tests of allelism among stable, independent *rfl* mutants that conditioned obvious lethal-kernel phenotypes. Crosses between CMS-S plants heterozygous for *rfl* and a nonrestoring allele are predicted to produce ears segregating 1:1 for aborted and normal seeds if the two mutants are alleles of the same gene, and ears with a full set of normal kernels if the two mutants affect different genes.

### Placement of restorer alleles on chromosomes

The *waxy1* (*wx1*)-marked reciprocal translocation series was used to test restorer alleles recovered in screens 1 and 2 for linkage with the long arm of chromosome 2 (2L), the known location of *Rf3* (Laughnan and Gabay 1978), *rfl1* (formerly *RfIII*), and *rfl2* (formerly *RfVI*) (Gabay-Laughnan *et al.* 1995). The translocation series was obtained from the Maize Genetics Cooperation Stock Center, Urbana, IL. The translocation lines employed in our studies do not carry restorer alleles for S cytoplasm. The use of *wx1*-marked reciprocal translocations to ascertain the chromosome location of restorer alleles was previously described (Laughnan and Gabay-Laughnan 1994). Briefly, CMS-S plants carrying an unplaced restorer allele are crossed with nonrestoring pollen from plants carrying a *wx1*-marked translocation. CMS-S plants heterozygous for a restorer exhibit 50% aborted pollen grains due to gametophytic fertility restoration (Buchert 1961). Maize plants heterozygous for a reciprocal translocation also exhibit 50% pollen abortion (Patterson 1994). CMS-S plants heterozygous for both a restorer allele and a reciprocal translocation exhibit 75% aborted pollen. These are the plants of interest that were crossed as pollen parents onto *wx1/wx1* tester plants. The proportion of waxy kernels on the resulting ear is a function of the recombination between the restorer and *wx1*.

### Immunoblotting of pollen proteins

Pollen was recovered from CMS-S plants heterozygous for restoring and nonrestoring alleles, and the starch-filled, restored pollen was physically separated from the collapsed, nonrestored pollen by sucrose density gradient centrifugation, as described by Wen and Chase (1999). Starch-filled, restored pollen pellets descend through 70% sucrose, whereas the nonrestored pollen, which collapses prior to appreciable starch accumulation, floats. For this reason, the restored and nonrestored pollen samples reflect distinctly different stages and programs of pollen development, and do not constitute developmentally comparable samples. Pollen recovered from N-cytoplasm Mo17 tassels was also pelleted through 70% sucrose to recover comparable developmental stages of control pollen for molecular comparisons, although it must be noted that these controls contain a genetically distinct cytoplasm. Pollen pellets of 0.25 g were frozen and stored at −80° until protein extraction. Total detergent-soluble proteins were extracted from frozen pollen pellets, fractionated by denaturing gel electrophoresis, transferred to nitrocellulose, immunodetected, and imaged as described in File S1. The primary antibodies used in this work are described in Table S1 in File S1.

### Data availability

The ccB73 UniformMu stock and stocks carrying the stably inherited *rfv* and *rfl* alleles are available by request to the corresponding author. File S1 contains all supplemental materials, including the detailed methods used to create our mutant screening populations (Figures S1–S3 in File S1), a detailed description of the antibodies used for immunoblotting (Table S1 in File S1), replication data for immunoblotting experiments (Figures S4 and S5 in File S1), seed abortion frequency data (Tables S2 and S3 in File S1), and recombination data for chromosome 2-linked restorer genes (Table S4 in File S1).

## Results

### Restorer mutations isolated from Ac-Ds and En/Spm transposon-active backgrounds

#### Mutation frequency:

**Figure 1:**
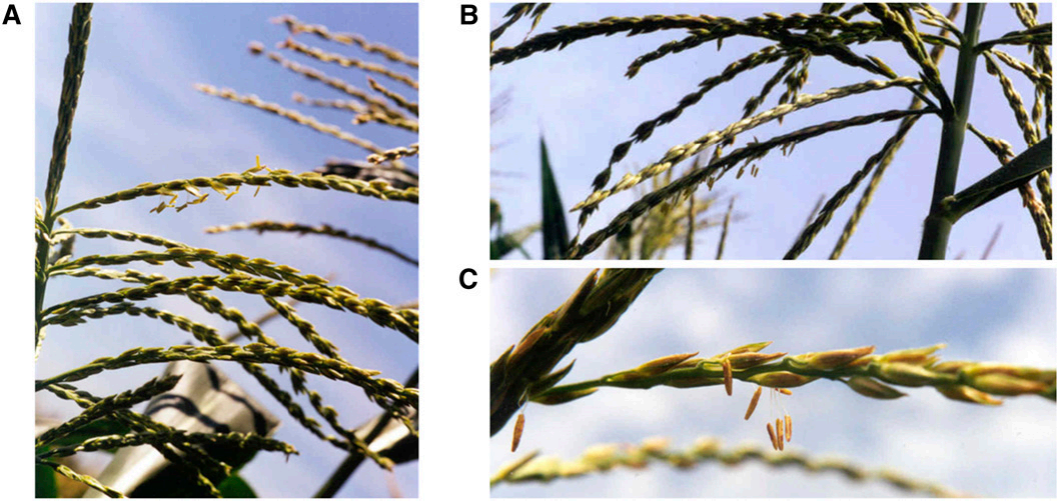
Tassel sectors of pollen fertility leading to the recovery of new restorer alleles. Pictures of pollen-fertile tassel sectors on cytoplasmic male sterility type S maize plants were taken during screen 1. Pollen from these sectors led to the recovery of (A) rfv*-99-1139, (B) rfv*-99-1181, and (C) rfv*-99-23.

In the screen of 1241 progeny plants carrying *Ac-Ds* (mutant screen 1), 12 plants with tassel fertility were identified and crossed as pollen parents onto CMS-S male-sterile tester plants. This represented a rate of mutation of just under 1%. Although tassel sectors were small (Figure 1), 10 of these crosses were successful and the progeny were analyzed for pollen fertility. Three of the new mutants shed little pollen, while seven had good to excellent pollen shed. Eight mutants were successfully crossed onto the CMS-S W64A and/or CMS-S Mo17 inbred nuclear backgrounds for continued analysis. In the screen of 1239 *En/Spm* plants (mutant screen 2), 27 plants with tassel fertility were identified and crossed as pollen parents onto CMS-S tester plants. These crosses produced 22 families that were grown and examined for pollen fertility. Of these, two did not show any evidence of fertility restoration, seven shed only small amounts of pollen, and the remaining 13 had fair to good pollen shed. The latter 13 restorers were crossed into the CMS-S W64A and/or CMS-S Mo17 inbred nuclear backgrounds for continued analysis. Thus, just over 1% of the putative *En/Spm*-induced mutations represented alleles producing adequate restoration. The eight screen 1 and 13 screen 2 restorer mutants that were successfully carried forward into CMS-S W64A or CMS-S Mo17 are listed in Table 2.

**Table 2:** Restorer mutants recovered from *Ac/Ds* and *En*(*Spm*) transposon-active, CMS-S maize stocks

Allele	Transposon Source	Stable Restoration in Mo17-S*^a^*
rfv*-99-23	*a1-m4* (*Ac/Ds*)	—
rfv*-99-50	*a1-m4* (*Ac/Ds*)	—
rfl*-99-78	*a1-m4* (*Ac/Ds*)	—
rfl*-99-114 (*rfl2-99-114*)	*a1-m4* (*Ac/Ds*)	+
rfv*-99-1139	*a1-m4* (*Ac/Ds*)	—
rfl*-99-1151-9	*a1-m4* (*Ac/Ds*)	—
*rf?**^b^* *-99-1157	*a1-m4* (*Ac/Ds*)	—
rfv*-99-1181	*a1-m4* (*Ac/Ds*)	+
rfv*-00-3305	*a1-m1*(*En/Spm*)	+
rfv*-00-3333	*a1-m1*(*En/Spm*)	—
rfv*-00-3336	*a1-m1*(*En/Spm*)	+
rfv*-00-3337-6	*a1-m1*(*En/Spm*)	—
rfv*-00-3337-8	*a1-m1*(*En/Spm*)	—
rfv*-00-3356	*a1-m1*(*En/Spm*)	±
rfv*-00-3362	*a1-m1*(*En/Spm*)	±
rfv*-00-3364-2	*a1-m1*(*En/Spm*)	—
rfv*-00-3364-9	*a1-m1*(*En/Spm*)	±
rfv*-00-3365	*a1-m1*(*En/Spm*)	—
rfl*-00-3376	*a1-m1*(*En/Spm*)	±
rfl*-00-3379	*a1-m1*(*En/Spm*)	+
rfl*-00-9050	*a1-m1*(*En/Spm*)	+

a Progeny after one or two backcrosses of restored pollen onto a Mo17-S seed parent were: all pollen sterile (−); segregating predominantly pollen-sterile plants (±); or segregating predominantly pollen-fertile plants (+).

b ? indicates that the effect of the allele on seed phenotype could not be ascertained with certainty.

#### Mutant classification:

The new restorer alleles were analyzed for viability of the homozygote on the basis of seed set on ears produced by self- or sib-crosses of CMS-S plants heterozygous for a new restorer allele. Four of the eight screen 1 restorers were homozygous viable (*rfv*); the self- or sib-crosses produced ears with full seed set. Three were homozygous lethal (*rfl*), with the self- or sib-crosses producing ears segregating ∼50% aborted or missing kernels, and one could not be classified with certainty. Ten of the 13 screen 2 restorers were *rfv* mutants and three were *rfl* mutants. Screens 1 and 2 therefore predominantly produced mutants of the *rfv* class.

#### Mapping and tests of allelism:

**Figure 2:**
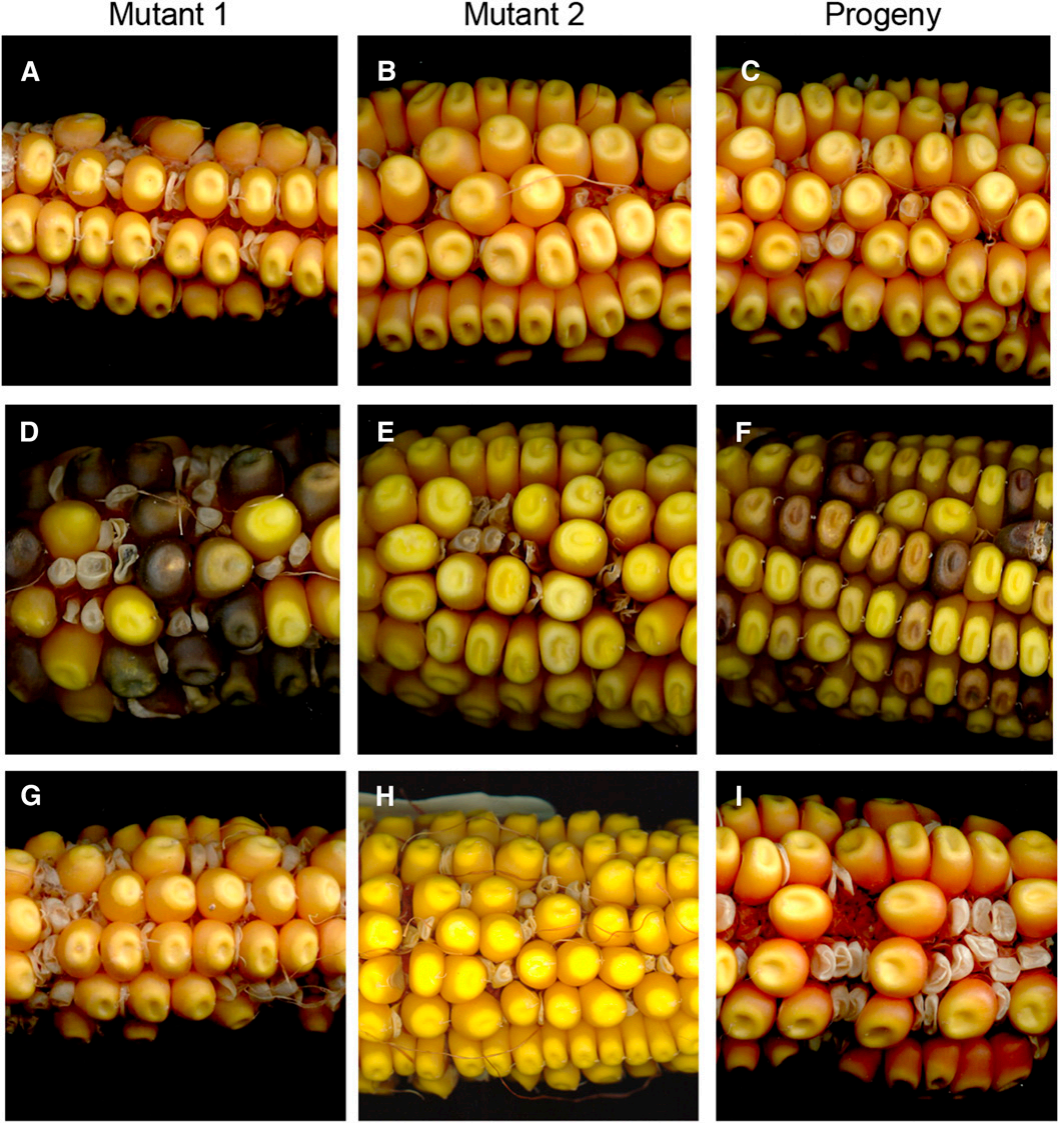
Tests of allelism among rfl* mutants. (A–C) A positive test of allelism based on the homozygous-lethal kernel phenotypes of (A) *rfl2-1* and (B) rfl*-99-114, which when crossed (C), failed to complement and restore normal seed set. (D–F) A negative test of allelism based on the homozygous-lethal kernel phenotypes of (D) rfl*-04-230 and (E) rfl*-04-229, which when crossed (F), complemented to restore full seed set. (G–I) A positive test of allelism based on the homozygous-lethal kernel phenotypes of (G) *rfl2-99-114* and (H) rfl*-06-78, which when crossed (I), failed to complement and restore normal seed set.

The *wx1*-marked reciprocal translocation series was used to test each of the new restorer alleles for linkage with chromosome 2L, the known location of three different restorer loci: *rf3*, *rfl1* (formerly *RfIII*), and *rfl2* (formerly *RfVI*). This strategy located rfv*-99-1181, rfl*-00-3379, and rfl*-99-114 on this chromosome arm. The clear mutant kernel phenotypes associated with *rfl2-1* and rfl*-99-114 provided the basis for a positive test of allelism between these chromosome 2L-linked mutants (Figure 2, A–C). Crossing the two mutants produced ears segregating ∼50% mutant and 50% normal kernels, as observed for ears produced by self- or sib-crosses of each mutant (Tables S2 and S3 in File S1). The two mutants were therefore allelic, and the rfl*-99-114 mutant was renamed *rfl2-99-114*.

In the absence of a seed phenotype, the genetic relationship between the two fertility-restoring alleles *Rf3* and rfv*-99-1181 was examined by determining the genetic distance between these two loci on chromosome 2L. CMS-S plants having *Rf3* and Rfv*-99-1181 on one chromosome, with *rf3* and rfv*-99-1181 on the homologous chromosome (heterozygous for each of the two restorer alleles *Rf3* and rfv*-99-1181), were pollinated with an N-cytoplasm nonrestoring plant. The resulting progeny were grown, and the frequency of pollen-sterile progeny was determined. Pollen-sterile progeny are the CMS-S rf3-Rfv*-99-1181 recombinant class, and the frequency of these recombinants represents half of the recombination distance between the two restorers. The CMS-S Rf3-rfv*-99-1181, double restorer recombinant class cannot be phenotypically distinguished from the nonrecombinant progeny that carry a single restoring allele. Four pollen-sterile recombinants recovered from 488 progeny demonstrated 1.6 cM separation between these loci on chromosome 2L. This strategy also demonstrated recombination distances of 19.8 cM between *Rf3* and *rfl1-1*, 21.4 cM between *Rf3* and *rfl2-1*, and 2.2 cM between *rfl1-1* and *rfl2-99-114*, defining two clusters of restorers separated by ∼20 cM on chromosome 2L (Table S4 in File S1). Other screen 1 and screen 2 restorers not linked to chromosome 2L remain unmapped.

#### Genetic instability of restorers arising in transposon-active lines:

Of the 21 restorer mutations carried forward from screens 1 and 2, 15 were genetically unstable (Table 2). Male-sterile progeny plants were recovered at frequencies of 57–100% after crossing CMS-S plants carrying these restorer alleles as pollen parents (Table 3). This was unexpected because in S cytoplasm, pollen function requires the presence of a restoring allele that should be transmitted to all progeny. Crosses were performed with eight independent screen 2 mutants to determine whether this genetic instability was the result of suppressible mutations that depend upon the presence of an autonomous transposable element for the mutant phenotype to be expressed (Table 3). Male-sterile progeny were recovered regardless of the presence or absence of autonomous *Spm* in the CMS-S seed parent. Moreover, once fertility restoration was lost, the introduction of autonomous *Spm* through a normal-cytoplasm pollen parent did not effectively reestablish fertility restoration (Table 3). The genetic instability of these restoring alleles made further genetic and phenotypic studies of these mutants largely unfeasible, and prompted additional mutant screens.

**Table 3:** Effects of autonomous *Spm* on the unstable restorer alleles

	Pollen-Fertile Progeny/Total Progeny			
Allele [Family]	Cross 1	Cross 2	Cross 3	Cross 4
	CMS-S rf*	CMS-S *Spm*	CMS-S	CMS-S rf* -off
	Self	X	X	X
		CMS-S rf*	CMS-S rf*	N *Spm*
rfv*-00-3337-6 [1]	3/12	1/13	0/13	0/14
rfv*-00-3337-6 [2]	1/13	1/12	4/13	2/14
rfv*-00-3337-6 [3]	0/11	4/12	2/14	
rfv*-00-3337-6 [4]	6/13	2/13		
rfv*-00-3337-8 [1]	10/14	7/13	2/8	0/11
rfv*-00-3337-8 [2]	3/11	0/12	0/10	1/12
rfv*-00-3337-8 [3]				1/12
rfv*-00-3356 [1]	6/11	0/9	2/12	0/11
rfv*-00-3356 [2]	5/12	2/11		0/13
rfv*-00-3356 [3]	8/11	3/13		
rfv*-00-3362 [1]	6/12	6/14	3/12	1/12
rfv*-00-3362 [2]	9/12	2/11	4/12	3/13
rfv*-00-3362 [3]	11/14	2/11	2/13	
rfv*-00-3362 [4]	7/9			
rfv*-00-3364 [1]	9/11	3/13	2/14	0/13
rfv*-00-3364 [2]	5/13	3/15	1/13	2/13
rfv*-00-3364 [3]				0/13
rfl*-00-3376 [1]	5/14	0/12	1/12	0/13
rfl*-00-3376 [2]	5/11	1/11	2/10	1/14
rfl*-00-3376 [3]	10/12			

CMS-S, cytoplasmic male sterility type S.

### Restorer mutations recovered from Mu transposon-active backgrounds

#### Mutation frequency:

In screen 3, a ccB73 UniformMu transposon resource, initially developed to screen for mutant kernel phenotypes, was explored as a source of CMS-S fertility restorers. Initially, ccB73 UniformMu plants were tested to ensure that a restorer allele for CMS-S was not uniformly present in this background. CMS-S Mo17 plants were pollinated with ccB73 UniformMu *Mu*-active plants. When two families of 30 progeny each were examined for pollen fertility, each family included 29 pollen-sterile and one pollen-fertile plant. The predominantly pollen-sterile progeny indicated that fertility restorers were not uniformly present in the ccB73 UniformMu genetic background. The two exceptional pollen-fertile progeny plants were successfully crossed as pollen parents onto CMS-S Mo17 ears. The resulting progeny were pollen-fertile and were carried forward as new restorer mutants rfl*-04-229 and rfl*-04-230.

Mutant screen 3 subsequently utilized the ccB73 UniformMu material as a source of additional restorer mutants by exploiting the observation that restoration of male fertility is often associated with seed lethality. Twenty-two ccB73 UniformMu ears segregating for seed lethality were screened for the presence of *rfl* alleles. Plump, stable bronze-pigmented (*Mu*-inactive) kernels were grown from these ears, and the resulting plants were crossed as pollen parents onto pollen-sterile CMS-S Mo17/B73 hybrid plants. Eight of the resulting 22 families included individuals that shed functional pollen. When these individuals were crossed as pollen parents to CMS-S Mo17/B73 pollen-sterile plants, progeny producing functional pollen were recovered in each case. Including the two mutants recovered from our first crosses between CMS-S Mo17 and ccB73 UniformMu, screen 3 yielded 10 independent restorer mutants (Table 4).

**Table 4:** Restorer mutants recovered following pollination of CMS-S plants with ccB73 UniformMu

Allele*^a^*	Stable Restoration in CMS-S Mo17/B73
rfl*-04-229	+
rfl*-04-230	+
rfl*-06-73-11	+
rfl*-06-76	+
rfl*-06-78 (*rfl2-06-78*)	+
rfl*-06-81	+
rfl*-06-85	+
rfv*-06-86	+
rfv*-06-88	+
rfv*-06-89	+

Progeny after one or two backcrosses of restored pollen onto a CMS-S Mo17/B73 hybrid seed parent were: all pollen sterile (−); segregating predominantly pollen sterile plants (±); or segregating predominantly pollen-fertile plants (+).

a Pollen parents were grown from plump, stable bronze-colored kernels on ccB73 UniformMu ears segregating for mutant kernels.

#### Mutant classification:

**Figure 3:**
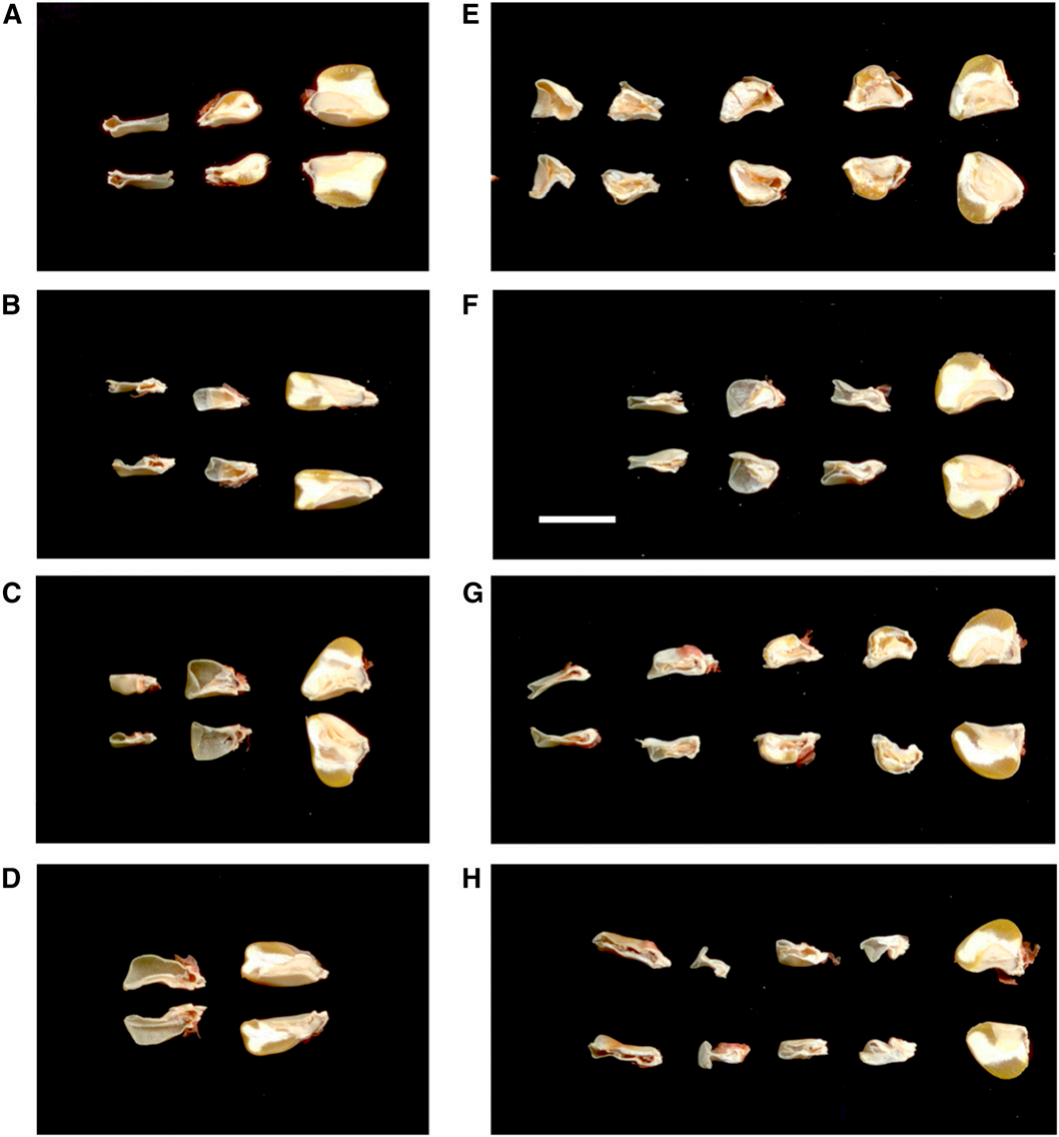
Variable seed-lethal phenotypes conditioned by *rfl* mutants. Scanned images of longitudinally split kernels that were isolated from a single, self-pollination progeny ear of a cytoplasmic male sterility type S plant heterozygous for mutant (A) *rfl2-99-114*, (B) *rfl2-06-78*, (C) rfl*-04-229, (D) rfl*-04-230, (E) rfl*-06-73, (F) rfl*-06-76, (G) rfl*-06-81, and (H) rfl*-06-85. In each panel, a nonmutant sibling kernel is shown on the right with representative mutant kernels ranging from defective kernel to empty pericarp phenotypes shown to the left. Scale bar, 1 cm.

Self-pollinations of CMS-S plants heterozygous for each of the screen 3 mutants demonstrated that seven were rfl* mutants. With one exception, the self-pollinated ears segregated ∼50% mutant kernels (Table S2 in File S1). The mutant kernels on these ears generally demonstrated a gradation of phenotypes ranging from empty pericarp (emp) to defective kernel (dek), with some vestiges of endosperm and embryo (Figure 3). For one mutant (rfl*-06-85), the self-pollinated ears were well set, but routinely segregated only 15%, rather than the expected 50%, lethal kernels. However, examination of pollen samples from 10 viable progeny revealed no plants that were homozygous for fertility restoration. In the absence of selection against the restoring allele on the maternal side, this class should comprise 50% of the progeny from a CMS-S rfv*/Rfv* self- or sib-pollination. The rfl* designation was therefore retained for this mutant. Three of the 10 independent restorer mutants isolated from screen 3 were rfv* mutants, as demonstrated by the full seed set on self-pollinated ears (data not shown).

#### Mapping and allelism:

Seed-lethal phenotypes were exploited for tests of allelism among the seven rfl* mutants recovered from screen 3. The *rfl2-1* and *rfl2-99-114* alleles were also included in this effort (Table 5). Pairwise crossing among all of the mutants provided genetic evidence that most were nonallelic. Complementation of nonallelic mutants was demonstrated by the full seed set on the progeny ears. The example of a cross between mutants rfl*-04-229 and rfl*-04-230 is shown in Figure 2, D–F. The only new positive allelism tests were those between *rfl2-1* and rfl*-06-78, and between *rfl2-99-114* and rfl*-06-78, renamed *rfl2-06-78* (Figure 2, G–I and Table S3 in File S1). The seven *rfl* mutants recovered from the screen of 24 ccB73 UniformMu families therefore defined six new *rfl* loci.

**Table 5:** Tests of allelism among stable *rfl* mutants based on seed phenotype

Mutant*^a^*	*rfl2-1*	*rfl2-99-114-7*	rfl*-04-229	rfl*-04-230	rfl*-06-73-11	rfl*-06-76-2	rfl*-06-78-6	rfl*-06-81-7	rfl*06-85-2
*rfl2-1*	X	Yes	NP	NP	No	No	Yes	NP	NP
*rfl2-99-114-7*	Yes	X	No	No	No	No	Yes	NP	No
rfl*04-229	No	No	X	No	No	No	No	No	No
rfl*04-230	No	No	No	X	No	No	No	NP	No
rfl*06-73-11	NP	NP	NP	No	X	No	No	NP	NP
rfl*06-76-2	NP	NP	No	No	No	X	NP	No	NP
*rfl2-06-78-6*	NP	Yes	No	No	No	No	X	No	No
rfl*06-81-7	No	No	NP	No	No	NP	NP	X	No
rfl*06-85-2	No	NP	NP	NP	No	No	No	NP	X

X, self-combination; Yes, mutants are allelic; NP, cross not performed or progeny ears not recovered; No, mutants are not allelic.

a Pollen-parent restorer alleles are indicated in column headings and seed-parent restorer allele is indicated in column 1.

### Molecular features of restored pollen

We hypothesize that the *rfl* mutants restore pollen fertility to CMS-S pollen by disrupting mitochondrial functions that are not essential to pollen function, but that are required for seed development. This hypothesis was investigated by targeted immunoblotting of mitochondrial respiratory proteins in restored CMS-S pollen, as compared to N-cytoplasm pollen without restorer alleles, to determine whether distinctive mitochondrial protein phenotypes accompanied fertility restoration by *rfl* or *rfv* mutants. Sucrose density gradient centrifugation was used to separate nonrestored CMS-S pollen, collapsed at the early bicellular stage, from starch-filled restored pollen at the late bicellular stage. Collapsed and restored CMS-S pollen were not developmentally comparable samples. Starch-filled, N-cytoplasm pollen without restorers was prepared for comparison to the restored CMS-S pollen, although different mitochondrial genotypes complicated these comparisons.

**Figure 4:**
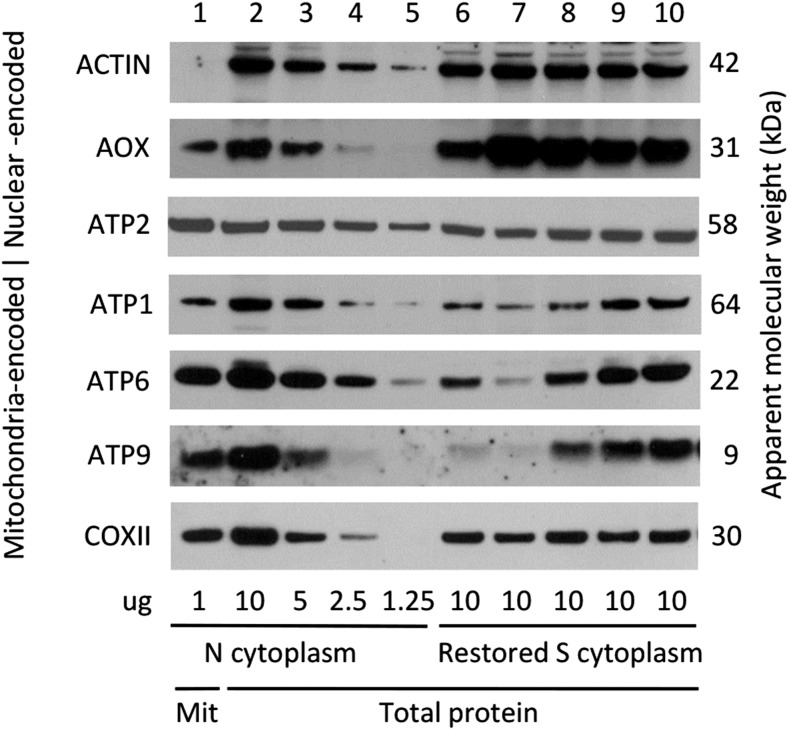
Mitochondrial protein accumulation in developing N-cytoplasm pollen and CMS-S pollen carrying homozygous-viable restorer alleles. Proteins were immunodecorated and detected following denaturing gel electrophoresis. Sample 1 contained 1 μg of protein extracted from N-cytoplasm Mo17 pollen mitochondria. Samples 2–5 contained 10, 5, 2.5, and 1.25 μg of N-cytoplasm Mo17 pollen total protein extract, respectively. Samples 6–10 contained 10 μg of total protein extract from S-cytoplasm pollen restored by *Rf3*, *rfv1-1*, rfv*-06-86, rfv*-06-88, and rfv*-06-89. The proteins detected are labeled on the left of each panel, with apparent molecular weights on the right. AOX, alternative oxidase; ATP1, ATP2, ATP6, and ATP9, ATP synthase subunits 1, 2, 6, and 9, respectively; CMS-S, cytoplasmic male sterility type S; COXII, cytochrome oxidase subunit 2.

**Figure 5:**
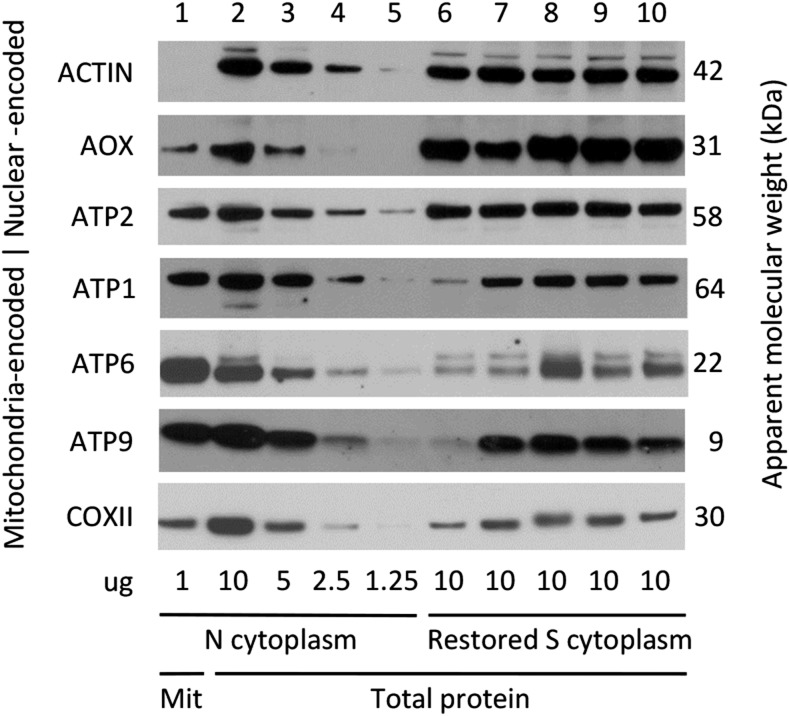
Mitochondrial protein accumulation in developing N-cytoplasm pollen and CMS-S pollen with rfl* restorers. Proteins were immunodecorated and detected following denaturing gel electrophoresis. Sample 1 contained 1 μg of protein extracted from N-cytoplasm Mo17 pollen mitochondria. Samples 2–5 contained 10, 5, 2.5, and 1.25 μg of total protein extract from N-cytoplasm Mo17 pollen, respectively. Samples 6–10 contained 10 μg of total protein extract from S-cytoplasm pollen restored by rfl*-06-73, rfl*-06-76, *rfl2-06-78*, rfl*-06-81, and rfl*-06-85, respectively. The proteins detected are labeled on the left of each panel, with apparent molecular weights on the right. AOX, alternative oxidase; ATP1, ATP2, ATP6, and ATP9, ATP synthase subunits 1, 2, 6, and 9, respectively; CMS-S, cytoplasmic male sterility type S; COXII, cytochrome oxidase subunit 2.

Two mitochondrial protein features distinguished the restored CMS-S pollen from comparably staged N-cytoplasm pollen without restorers (Figure 4 and Figure 5). First, the abundance of nuclear-encoded, mitochondrial alternative oxidase (AOX) was increased relative to that of the mitochondria-encoded cytochrome oxidase subunit II (COXII) in the restored CMS-S samples. Second, the abundance of mitochondria-encoded ATP1 was decreased relative to that of the nuclear-encoded ATP synthase subunit 2 (ATP2) in the restored CMS-S samples. These features were observed in both *rfl*- and *rfv*-restored pollen and were confirmed in a set of biological replicate samples (Figures S4 and S5 in File S1). Additionally, while the abundance of nuclear-encoded ATP2 was consistent among the N-cytoplasm and restored CMS-S pollen samples, reduced accumulation of mitochondria-encoded ATP6 and/or ATP9 was observed in several restored CMS-S pollen genotypes. This feature was observed for each of the *rfl* restorers examined, but was inconsistent between the *rfl2-06-78* replicates (Figure 5 and Figure S5 in File S1). Among the homozygous-viable restorers, this effect was seen in CMS-S pollen restored by *Rf3* or *rfv1-1*. In the latter case, the restored pollen accumulated only one-eighth of the ATP6 and ATP9 observed in the Mo17 N-cytoplasm pollen (Figure 4 and Figure S4 in File S1).

## Discussion

### Mutant screens for cloning restorer genes in maize

CMS-S maize presents a unique opportunity to screen for transposon-induced loss-of-function restorers to facilitate cloning by transposon tagging. This strategy differs from the conventional, targeted screening for loss-of-function mutants in a specific, dominant restorer gene. In contrast to the screen for a tagged *Rf2* allele conducted by Schnable and Wise (1994), in which populations of male-fertile maize plants were screened for male-sterile exceptions, we looked among populations of male-sterile plants for exceptional male fertility. One male-fertile plant in a field of male-sterile plants is much more easily observed than a single male-sterile plant in a field of male-fertile plants. Schnable and Wise (1994) detasseled the male-fertile plants to facilitate the identification of male-sterile exceptions. This labor-intensive approach was unnecessary in our system. Furthermore, the targeted transposon mutagenesis of a dominant restorer results in pollen sterility. By necessity, the mutation must be recovered through the female gamete, so the insertion event must occur early enough in plant development to be recovered through the ear. In our screen, restorers were successfully recovered from small pollen sectors. Finally, the untargeted strategy allowed by CMS-S resulted in the recovery of multiple independent restorer alleles in screens of ∼1000 plants, in contrast to the screening of several thousand plants required in a targeted screen.

### Genetic instability of restorer alleles

Although screens of CMS-S *Ac-Ds* and *En/Spm* populations initially recovered novel restorers at the rate of ∼1%, the majority of restorers recovered in these screens were unstable. An exception was the *rfl2-99-114* mutant discussed below. One possible explanation for mutant instability is the recovery of suppressible, transposon-insertion mutations, in which an insertion only conditions an altered phenotype in the presence of an autonomous element of the same transposable element family (Barkan and Martienssen 1991; Grant *et al.* 1990). However, the introduction of autonomous *Spm* into eight unstable screen 2 restorer mutant stocks did not restore fertility. An alternative explanation for restorer mutation instability might be that many of the screen 1 and screen 2 loss-of-function restoring alleles arose through epigenetic silencing events that did not persist for multiple generations. Regardless of the cause, the instability of most screen 1 and screen 2 mutants made further genetic and molecular characterization impractical. This, along with the use of *Mu* transposons for the cloning of numerous maize genes (McCarty *et al.* 2013; Williams-Carrier *et al.* 2010), led us to develop and subsequently focus on the mutants from screen 3. These mutants proved to be much more stable, possibly because the development of ccB73 included selection against epigenetic instability, as observed in the anthocyanin pathway.

### Restorer mutants recovered from Mu transposon-active lineages

W22 UniformMu is a powerful maize genetic resource for forward (Hunter *et al.* 2014) and reverse (McCarty *et al.* 2013) genetic screens. Here, a parallel resource was established in the ccB73 background. B73 is known to maintain rather than restore pollen fertility in S cytoplasm, hence ccB73 UniformMu was also a promising source of new *rfl* mutants. In screen 3, ccB73 UniformMu families segregating lethal-kernel mutants were selected for subsequent *rfl* mutant screening. For this reason, the frequency of mutants recovered in screen 3 cannot be compared to the frequencies observed in screens 1 and 2. Nevertheless, the screen 3 strategy was highly efficient. First, the preliminary screen of two CMS-S Mo17 × ccB73 UniformMu (*Mu*-active) progenies, performed to test whether CMS-S restorers were uniformly present in the ccB73 UniformMu background, recovered two new nonallelic restorers: rfl*-04-229 and rfl*-04-230. Subsequently, crosses of plants from 22 ccB73 UniformMu (*Mu*-inactive) families segregating for lethal-kernel mutants yielded three independent *rfv* mutants and five nonallelic *rfl* mutants. We did not investigate whether the *rfl* mutants recovered were allelic to the original lethal-kernel mutants segregating on the ears sourced for screen 3. Nevertheless, through the recovery of two *rfl* mutants from the preliminary test for restorer activity and the recovery of *rfv* mutants from the subsequent screen, we see that prior selection of lethal-kernel mutants is not essential for the recovery of new restorer mutants from the ccB73 UniformMu resource.

### Mapping and allelism of new restorers

Our study uncovered additional restorer mutants mapping to chromosome 2L, where the native *Rf3* (Kamps and Chase 1997; Laughnan and Gabay 1978; Xu *et al.* 2009), spontaneous *rfl1-1* (formerly *RfIII*) (Gabay-Laughnan *et al.* 1995; Wen *et al.* 2003), and spontaneous *rfl2-1* (formerly *RfVI*) (Gabay-Laughnan *et al.* 1995) are located. Recombination distances between *Rf3* and rfv*-99-1181, *Rf3* and *rfl1-1*, *Rf3* and *rfl2-1*, and *rfl1-1* and *rfl2-99-114* demonstrated two clusters of restorer gene loci an estimated 20 cM apart on 2L: one including the *rf3* and rfv*-99-1181 loci and a second that included the *rfl1* and *rfl2* genes.

The obvious lethal-kernel phenotypes associated with many of the *rfl* mutants provided a convenient means to test for allelic mutations. Pairwise crosses among nine such mutants, eight recovered in our screens and the spontaneous *rfl2-1*, demonstrated that rfl*-99-114 (now *rfl2-99-114*) and rfl*06-78 (now *rfl2-06-78*) were allelic to each other and also to *rfl2-1*. All pairwise crosses among these mutants gave consistent, positive tests of allelism. All other mutant combinations resulted in genetic complementation defining them as nonallelic. Given that the current collection of spontaneous *rfl* mutants includes 41 nonallelic mutants (S. Gabay-Laughnan, unpublished data), complementation (nonallelism) among nine mutants would be expected. However, the recovery of independent *rfl2* alleles by spontaneous mutation (*rfl2-1*) and in two of three transposon screens was unexpected. This finding demonstrated that the *rfl2* locus was unstable and frequently altered to create an *rfl2* allele, possibly by mechanisms other than transposon insertion, as discussed below.

In the case of *rfv* mutants, there is no functional test of allelism equivalent to that provided by the lethal-kernel phenotype. If two mutants map to different regions of the genome, allelism can be excluded. However, if the mutants map to the same region of the genome, they can only be tested to see if they can be separated by recombination, for example, as for the *Rf3* and rfv*-99-1181 restorers. At this time, the allelic relationships of rfv*-06-86, rfv*-06-88 and rfv*-06-89 remain unresolved.

### Loss-of-function and gain-of-function restorer alleles

While dominance relationships cannot be determined in haploid pollen, such relationships can be resolved in pollen derived from tetraploid maize. Studies of this type demonstrate that restoring alleles can be dominant, as in the case of *Rf3* (Kamps *et al.* 1996), or recessive, as in the case of *rfl1-1* (Wen *et al.* 2003). The homozygous-lethal phenotype conditioned by *rfl1-1* is consistent with its recessive nature, and with our model in which *rfl* mutations restore fertility to CMS-S pollen by interfering with mitochondrial functions that are expendable in the pollen but essential to seed development. While this model presents a straightforward explanation for the nature of the *rfl* mutations, other more complex scenarios are possible, and kernel phenotypes need not always reflect the dominant or recessive nature of a restorer allele. An *rfv* allele might result from a recessive, loss-of-function mutation in a pollen-expressed gene that has a functional paralog in the developing kernel. The unstable *rfl2* locus might be the result of clustered genes encoding PPR proteins. Such clusters can undergo recombination to produce novel *ppr* genes that gain the function of fertility restoration (Gaborieau *et al.* 2016; Melonek *et al.* 2016). Such a recombination event could, at the same time, delete a *ppr* gene having an essential function in seed development. The result would be a tightly linked dominant restorer and recessive lethal-kernel mutation. Alternatively, a recombination event could simply delete an essential *ppr* gene to create a straight-forward *rfl* mutation. In either recombination scenario, the *rfl2* mutant alleles would not result from transposon insertions, and map-based cloning (Gallavotti and Whipple 2015) would be the logical approach for the identification and characterization of the *rfl2* locus.

### Mitochondrial functions in pollen development

Two mitochondrial protein features identified here generally distinguished restored CMS-S pollen from comparably staged N-cytoplasm pollen without restorers. First, the relative abundance of AOX and COXII respiratory proteins was reversed such that AOX was more abundant in restored CMS-S pollen. Second, the relative abundance of ATP1 and ATP2 was reversed such that ATP1 was less abundant in restored CMS-S pollen. Because these features are common to most or all of the restored genotypes, they might reflect differences in mitochondrial genotypes rather than the effects of fertility restoration. Unfortunately, it is not possible to compare the molecular features of comparably staged CMS-S and CMS-S *rfl* pollen. CMS-S pollen is collapsed at the young bicellular stage, but it cannot be effectively separated from CMS-S *rfl* pollen until the restored pollen has reached the late starch-filling stage. In the case of the *rfv* restorers, the creation of near-isogenic *rfv/rfv* CMS-S and *rfv/rfv* N-cytoplasm genotypes will allow the effects of cytoplasm and restorer to be distinguished.

Individual restorer mutants were, however, associated with distinctive mitochondrial protein losses that demonstrated a surprising plasticity of maize pollen function with respect to respiratory protein composition. Notably, pollen restored by *Rf3* contained little ATP9, and pollen restored by *rfv1-1* was depleted of ATP6 and ATP9. Pollen restored by *rfl* alleles accumulated reduced levels of one or both proteins. Although pollen development is considered to be an energy-demanding process (Warmke and Lee 1978), CMS-S maize pollen carrying these restoring alleles was capable of effecting fertilization, despite lacking a conventional complement of mitochondrial respiratory proteins. In these cases, the accumulation of AOX might be critical to pollen function. AOX is a terminal oxidase that bypasses two of three phosphorylation sites in the mitochondrial respiratory chain (Vanlerberghe 2013). The mitochondrial accumulation of AOX is upregulated under conditions of respiratory, biotic, and abiotic stress, in what is considered to be a major mechanism of metabolic homeostasis (Rogov and Zvyagilskaya 2015; Saha *et al.* 2016; Vanlerberghe *et al.* 2013). The increased expression of maize genes encoding AOX proteins is seen in many of the lethal-kernel mutants associated with mitochondrial dysfunction (Cai *et al.* 2017; Chen *et al.* 2017; Wang *et al.* 2017; Xiu *et al.* 2016; Yang *et al.* 2017), and in all major organs of maize plants in which essential mitochondrial functions are compromised by nonchromosomal stripe mutations (Karpova *et al.* 2002). In restored CMS-S pollen, the AOX to COXII ratios demonstrated a greater potential for, and possibly reliance upon, the alternative pathway of respiration when function of cytochrome pathway and ATP synthase was compromised by the CMS-S mitochondrial genotype or by the mechanism of fertility restoration.

These observations are consistent with observations of pollen function in other species. The lily pollen tube, for example, adapts to and continues growth in the presence of mitochondrial respiratory inhibitors (Obermeyer *et al.* 2013; Rounds *et al.* 2010). In petunia and tobacco pollen, aerobic fermentation through a pyruvate dehydrogenase bypass comprised of pyruvate decarboxylase and aldehyde dehydrogenase is active during normal pollen tube growth (Gass *et al.* 2005; Mellema *et al.* 2002). This bypass has been hypothesized to support pollen fertility in maize (Tadege *et al.* 1999). The metabolic pathways utilized for pollen maturation and function in restored CMS-S maize require further investigation, and will provide additional insights into the mitochondrial requirements and alternative metabolic strategies that support pollen development and fertility.

### Mechanisms of fertility restoration

The mitochondrial events leading to CMS-S pollen collapse are not well understood, but aspects of mitochondrial gene expression are required to execute this phenotype. A 1.6-kb mitochondrial transcript encoding two novel open reading frames, *orf355* and *orf77*, is associated with the expression of CMS-S (Gabay-Laughnan *et al.* 2009; Matera *et al.* 2011; Zabala *et al.* 1997), and the mitochondrial C-to-U RNA editing process further modifies the *orf77* sequence to predict *orf17* (Gallagher *et al.* 2002). No translation products of the 1.6-kb RNA have as yet been detected, so it is not clear whether the transcript itself or a protein product is responsible for the CMS-S phenotype. *Orf17* predicts a peptide highly similar to the C-terminal transmembrane domain of the mitochondrial ATP9 subunit (Gallagher *et al.* 2002). The association of several restorers with the reduced accumulation of this subunit supports models of CMS-S involving the expression of *orf17*. The loss of mitochondria-encoded respiratory proteins in restored CMS-S pollen indicates that compromised mitochondrial gene expression processes constitute a mechanism of fertility restoration. Such a mechanism could silence the expression of the CMS-S gene or genes at the expense of mitochondrial respiratory gene expression, but because of pollen metabolic flexibility, this restored pollen can still effect fertilization.

### Mitochondrial functions in seed development

We hypothesize that the seed-lethal phenotypes of the *rfl* mutants stem from mitochondrial dysfunction, potentially the result of the same compromised mitochondrial gene expression that rescued the CMS-S pollen. This hypothesis is consistent with other maize mutants that are known to disrupt mitochondrial gene expression and to condition seed lethality. A recent forward genetic screen investigated 12 maize seed mutants and identified four that cosegregated with *Mu* insertions in genes predicting mitochondria-targeted proteins. These included three PPR proteins and a transcription termination factor (Hunter *et al.* 2014). Also in maize, numerous mutations in the broader class of *ppr* genes (those that are not members of the *restorer-of-fertility-like* class) have been reported to disrupt kernel development. These mutations commonly condition defects in specific plant mitochondrial C-to-U RNA editing events required for transcripts to encode correct protein sequences (Liu *et al.* 2013; Qi *et al.* 2017a; Sun *et al.* 2015; Wang *et al.* 2017; Yang *et al.* 2017), or cause defects in specific plant mitochondrial RNA splicing events (Cai *et al.* 2017; Chen *et al.* 2017; Qi *et al.* 2017b; Xiu *et al.* 2016; Yang *et al.* 2017) leading to the loss of mitochondrial translation products. Others affect the accumulation or translation of specific mitochondrial transcripts (Gutiérrez-Marcos *et al.* 2007; Lee *et al.* 2017; Manavski *et al.* 2012).

While not all maize seed-lethal mutants have a direct connection to mitochondrial dysfunction, those that do point to the importance of mitochondrial function in seed development. Embryos in kernels with mitochondrial defects are often observed to arrest at the transition stage. Mitochondria are abundant in the basal endosperm transfer layer (BETL) cells of nonmutant maize kernels (Becraft and Gutierrez-Marcos 2012), and defects in the development of the BETL cells are characteristic of mutants that compromise mitochondrial function. These observations led to models in which functional mitochondria are required for BETL cell differentiation and/or development, and in which functional BETL cells are needed for proper endosperm and perhaps embryo development (Chen *et al.* 2017; Chettoor *et al.* 2015; Manavski *et al.* 2012; Wang *et al.* 2017). Related to this point, *emp6/emp6* embryos, deficient in a mitochondrial plant organelle RNA recognition protein, fail to develop normally even in the presence of nonmutant endosperm (Chettoor *et al.* 2015), indicating that there are mitochondrial functions that are specific to embryo development.

Whether a seed-lethal mutation also conditions fertility restoration in S-cytoplasm pollen depends upon the expression pattern of the nonmutant form of the gene, whether there are paralogs with duplicate or overlapping functions and expression patterns, and whether the mutation affects mitochondrial processes central to the expression of CMS-S. The *emp4* mutant does not restore pollen fertility in S cytoplasm (S. Gabay-Laughnan, unpublished data), but this gene is not highly expressed in developing maize anthers (Gutiérrez-Marcos *et al.* 2007). By the same logic, not all restorers that compromise mitochondrial function condition seed lethality. In the case of *rfv1-1*, associated with depletion of key ATP synthase subunits, the function of paralogous genes might explain the viable homozygous mutant seed phenotypes.

### Conclusions

CMS-S maize presents a novel paradigm of fertility restoration that allows the recovery of multiple, nonallelic restorer mutations. The lethal-kernel phenotypes and pollen mitochondrial protein phenotypes conditioned by these mutants demonstrate that most differ fundamentally from the gain-of-function restorers that evolved in concert with other CMS systems. The UniformMu lines constitute a forward genetic resource that can be used to further expand the collection of CMS-S restorer mutations, and also to identify additional lethal-kernel mutants that can be examined for fertility restoration in a reverse-genetics approach. *Mu* insertion alleles provide a route to molecular cloning of multiple CMS-S restorer loci. Cloning, along with detailed molecular and cellular characterization of these mutants, will advance our understanding of S male sterility, and the roles of mitochondria in the development and function of the maize male gametophyte.

## Supplementary Material

Supplemental material is available online at www.g3journal.org/lookup/suppl/doi:10.1534/g3.117.300304/-/DC1.
