# Supplementary material for: Restorer-of-Fertility Mutations Recovered in Transposon-Active Lines of S Male-Sterile Maize
Source: G3 (Bethesda). 2017 Nov 22;8(1):291–302. doi: 10.1534/g3.117.300304 (PMC5765357; doi:10.1534/g3.117.300304)
Supplement: Supplementary file 1 [file 291FileS1.pdf]

Restorer-of-fertility mutations - File S1 - Supplemental materials

MATERIALS AND METHODS

Mutant Screens

***Activator-Dissociation* (*Ac-Ds*) populations:** We developed Mo17 *Ac-Ds* transposon-active stocks in both normal (N) and CMS-S cytoplasms as outlined in Figure S1. CMS-S Mo17 exhibits a very low rate of spontaneous reversion to pollen fertility (Gabay-Laughnan and Chase 2000). We therefore expected a low rate of background, spontaneous restorer mutations in the CMS-S Mo17-converted transposon stocks. The *a1-m4* stock (*Ds* inserted at *a1* on chromosome 3, *Ac* in chromosome 9) was obtained from Dr. M. G. Neuffer at the University of Missouri. The genotype of this stock is *a1-m4::Ds Sh2/a1-s Sh2; Ac/-*. N-cytoplasm Mo17 (*A1 Sh2/A1 Sh2*) ears were crossed with pollen from the *a1-m4* stock. The resulting F1 plants were again crossed as females with pollen from the *a1-m4* stock. Spotted kernels, indicative of an active *Ac*, on the resulting ears were used as the source of N-cytoplasm *a1-m4*. This stock, N-Mo17/*a1-m4*^{2} (N-cytoplasm Mo17/*a1-m4* F1 backcrossed with *a1-m4*), lacked restoring alleles for CMS-S and carried a *Ds* element at *a1* on chromosome 3 and an *Ac* element on chromosome 9. The S-cytoplasm Mo17 *a1-m4* stock was developed by crossing ears from a sterile CMS-S Mo17/H95 //Mo17^3 stock (Mo17/H95 F1 crossed recurrently by the Mo17 inbred line for three generations) with pollen from the original *a1-m4* stock. When the N-Mo17/*a1-m4* F1 was used as pollen parent onto ears of the CMS-S Mo17/*a1-m4*, spotted kernels segregated on the resulting ears. These spotted kernels were planted and the resulting pollen-sterile plants were crossed by either the N-cytoplasm Mo17/*a1-m4*^{2} stock or the original *a1-m4* stock to generate sufficient spotted kernels for a mutant screen. In mutant screen one, we screened 1,241 CMS-S *Ac-Ds*-carrying plants for new restorer mutants.

***Enhancer/Suppressor-mutator* (*En/Spm*) populations:** *En/Spm,* or for brevity, *Spm*, stocks were also developed in S and N cytoplasms (Figure S2). Dr. P. A. Peterson at Iowa State University supplied the *a1-m1* source with *I(dspm)* inserted at *a1* on chromosome 3 and autonomous *En*/*Spm* (also in chromosome 3S). The genotype of this stock was *a1-m1::I sh2 Spm Et/a1 Sh2* no *Spm et.* By selecting full (non-shrunken) spotted kernels, we maintained this stock as a heterozygote. By pollinating ears of these heterozygotes with N-cytoplasm Mo17 (*A1 Sh2* no *Spm/A1 Sh2* no *Spm*) we generated vigorous N-cytoplasm *A1 Sh2* no *Spm / a1-m1::I sh2 Spm* plants, but CMS-S Mo17 sterile plants matured too late to be crossed with pollen from the *a1-m1* stock. We therefore used early maturing CMS-S plants of the genotype ML-W64A/Oh545 or VG-W23/H109 in order to obtain *a1-m1* in S-type cytoplasm. These pollen-sterile plants were crossed by the *a1-m1* stock, the resulting plants were sterile, indicating that the *a1-m1* stock did not carry an S restorer. The sterile testcross plants (ML-W64A/Oh545//*a1-m1* or VG-W23/H109//*a1-m1*) were crossed either by the *a1-m1* stock or the *a1-m1*/Mo17 F1 to obtain kernels to screen for new restorers. Spotted kernels, indicative of an active *En/Spm*, from the resulting ears were planted producing 852 progeny plants that were screened for fertility. We also planted 581 kernels without spots from these same ears knowing that 2/3 of them would carry *En/Spm.* In mutant screen two, 1433 CMS-S plants (an estimated 1239 carrying *En/Spm*) were searched for restorer mutants.

B73 UniformMu populations: A B73 UniformMu resource, originally developed to study seed mutants, was investigated as a source of *Mu*-transposon-induced *rfl* mutants. The B73 version of UniformMu was developed as shown in Figure S3A, using a similar strategy as the W22 UniformMu population (Settles *et al.* 2004). A color-converted version of the B73 inbred

(ccB73) was created by crossing N-cytoplasm B73 with the University of Florida color-converted W22 stock. Plants grown from purple kernels were backcrossed to B73 for 8 cycles, selecting for purple kernels each generation. The autonomous *Mu* and *bz1-mum9* reporter allele were introduced into the ccB73 background from the BC_7S_1 generation of W22 UniformMu (Settles *et al*. 2004). Sixty *bz1-mum9*/*bz1-mum9* families were crossed with ccB73 to make an F1. Each pollen parent for the F1 was self-pollinated and scored visually for somatic *Mu* activity and presence of seed mutant phenotypes. F1 crosses from the upper 10% of *Mu*-active parents were selected for advancement to BC1 with a counter selection to remove seed mutants. The *Mu* score also selected for improved anthocyanin expression. Fifty-one BC1 families were then self-pollinated and crossed onto ccB73 ears for a similar selection of BC2 families. Additional generations of backcrossing to ccB73 with concomitant self-pollination to select for *Mu* activity and counter select for seed mutants created a ccB73 UniformMu resource generating ears that segregated for independent seed development mutants.

The ccB73 UniformMu resource was subsequently investigated as a source of *Mu* transposon-induced *rfl* mutants as outlined in Figure S3B. The ccB73 version of UniformMu was selected for this screen because B73 is known to maintain CMS-S pollen sterility. To confirm maintenance of pollen sterility in the ccB73 UniformMu background, CMS-S Mo17 was pollinated with ccB73 UniformMu plants grown from two ears that were not segregating seed mutants. Thirty progeny from each of the two resulting families were grown and examined for pollen fertility. The largely pollen-sterile families confirmed the absence of fertility restorers in the ccB73 UniformMu background, but two pollen-fertile exceptions (one in each family) were back crossed to CMS-S Mo17 and carried forward as new *rfl* candidates. Subsequently, plump

kernels from 22 ccB73 UniformMu ears segregating for independent, seed-lethal mutants were grown and the resulting plants were crossed to pollen-sterile CMS-S Mo17/B73 hybrid plants. In mutant screen three, 22 progeny families of 15 plants each (one family tracing back to each of the 22 ccB73 UniformMu ears) were grown and screened for pollen fertility.

Protein extraction and immunoblotting

Total, detergent-soluble proteins were extracted from frozen pollen samples. Pollen pellets of 0.15 g were mixed with 300 μL of 1X NuPAGE® LDS sample buffer (Thermo-Fisher Scientific Inc., Waltham, MA) containing 50 mM dithiothreitol (DTT) and 1.0 mM phenyl methyl sulfonyl fluoride (PMSF). The mixture was incubated at 70° for 10 minutes with intermittent vortex mixing. Insoluble materials were pelleted by two centrifugations at 12,000 x g for 10 min. The supernatants were recovered. An aliquot of each extract was diluted 1/50 in sterile distilled water and the dilution was used to determine the protein concentration in the 660 nm protein assay in the presence of ionic detergent compatibility reagent (Thermo-Fisher Scientific Inc.). Extracts were diluted to a concentration of 0.5 μg/μL, fractionated by electrophoresis through pre-cast NuPAGE® gels (Thermo-Fisher Scientific Inc.), transferred to nitrocellulose and decorated with primary antibodies followed by horseradish peroxidase (HRP)-conjugated secondary antibodies. The primary antibodies used in this work are described in Table S1, below. Replicate gels and blots were prepared so that blots did not have to be stripped to assay proteins of similar size. Chemiluminescent signals were generated by incubating the blots in SuperSignal™ West Pico chemiluminescent HRP substrate (Thermo Fisher Scientific Inc.) and detected by exposing the blots to X-ray film. Subsequently, the original extracts and a set of

biological replicate extracts were used to produce replicate blots that were imaged in a ChemiDoc ARS+ System (Bio-Rad Laboratories, Hercules, CA). Image Lab™ software (Bio-Rad Laboratories) was used to determine raw band volumes from the strongest exposures that did not contain saturated pixels. Within-sample ratios for AOX/COXII and for ATP1/ATP2 signals were computed and normalized to those of the N-cytoplasm Mo17 pollen control samples. Means and standard deviations for these ratios were calculated in Excel. One-tailed T values, also calculated in Excel, were used to test the statistical significance of protein ratio differences between restored CMS-S and N-cytoplasm Mo17 pollen samples.

LITERATURE CITED

Elthon, T. E., R. L. Nickels, and L. McIntosh, 1989 Monoclonal antibodies to the alternative oxidase of higher plant mitochondria. Plant Physiol. 89: 1311-1317.

Gabay-Laughnan, S. and C. D. Chase, 2000 Transposon tagging of nuclear genes that control mitochondrial gene expression. Maize Genet. Coop. Newslett. 74: 73.

Karpova, O. V., E. V. Kuzmin, T. E. Elthon, and K. J. Newton, 2002 Differential expression of alternative oxidase genes in maize. Plant Cell 14: 3271-3284.

Lu, B., and M. R. Hanson, 1994 A single homogeneous form of ATP6 protein accumulates in petunia mitochondria despite the presence of differentially edited *atp6* transcripts. Plant Cell 12: 1955-1968.

Luethy, M. H., A. Horak, and T. E. Elthon, 1993 Monoclonal Antibodies to the [alpha]- and [beta]-Subunits of the Plant Mitochondrial F1-ATPase. Plant Physiol. 101: 931-937.

Pring. D. R., H. V. Tang, C. D. Chase, and M. N. Siripant, 2006 Microspore gene expression associated with cytoplasmic male sterility and fertility restoration in sorghum. Sex. Plant Reprod. 19: 25-35.

Settles, A. M., S. Latshaw, and D. R. McCarty, 2004 Molecular analysis of high-copy insertion sites in maize. Nucleic Acids Res. 32: e54.

Table S1 Antibodies used for protein immunodetection

Target (kDa)[a] Genome[b]	Antigen	Antibody type[c]	Source or Citation
ACTIN 11 (42.6) N	*A thalinana* full length Actin-11 NP_187818.1	MMC	Agrisera AS10 702
AOX (34.0 & 36.0)[d] N	AOX purified from *S. guttatum*	MMC	Elthon *et al.* 1989
ATP1 (55.2) M	Maize mitochondria	MMC	Leuthy *et al.* 1993
ATP2 (52.2) N	Maize mitochondria	MMC	Leuthy *et al.* 1993
ATP6 (28.0)[e] M	Peptide: SPLDQFGIHPILDLN	RPC	Pring *et al.* 2006
ATP9 (7.6) M	Peptide: LEGAKLIGAGAAT	RPC	This study

COXII (29.7) M	Peptide: proprietary	RPC	Agrisera AS04 0530A

[a] The Protein Molecular Weight Calculator (http://www.bioinformatics.org/sms/prot_mw.html, accessed 07/14/2015) was used to predict the molecular weights of maize proteins based upon their predicted amino acid sequences minus, in the case of nuclear-encoded proteins, the predicted mitochondrial targeting sequences

[b] M, mitochondria encoded; N, nucleus encoded

[c] MMC, mouse monoclonal antibody; RPC, rabbit polyclonal antibody

[d] Observed molecular weights of maize AOX2 and AOX3 (Karpova *et al.*2002)

[e] Based upon the mature (processed) form of petunia ATP6 (Lu and Hanson 1994)

Table S2 Lethal-kernel phenotypes observed on self-pollinated ears of CMS-S plants heterozygous for a restoring allele

Restorer mutation	Progeny seed phenotype	
	n[a]	% aborted[b]
rfl2-1	2	52.4+2.7
rfl2-99-114	3	45.9+4.4
rfl-04-229*	6	38.9+10.2
rfl-04-230*	5	52.7+3.1
rfl-06-73*	3	47.2+2.7
rfl-06-76*	3	47.6+2.4
rfl2-06-78	3	40.9+10.8
rfl-06-81*	3	49.6+3.5
rfl-06-85*	4	14.9+1.2

[a] n, the number of progeny ears analyzed

[b] The mean % of aborted seeds (± standard deviation) per ear, determined by counting all normal (starch-filled) and aborted (empty, collapsed) seeds on each ear; ears contained 130-694 kernels; standard deviations were calculated at http://www.mathportal.org/calculators/statistics-calculator/standard-deviation-calculator.php (accessed 4/12/2017)

Table S3 Seed phenotype data for positive tests of allelism between *rfl* alleles

Seed parent restoring allele	Pollen parent restoring allele	Progeny seed phenotype	
		n[a]	% aborted[b]
rfl2-1	*rfl2-99-114*	1	49.0
rfl2-1	*rfl2-06-78*	1	44.0
rfl2-99-114	*rfl2-1*	1	31.5
rfl2-99-114	*rfl2-06-78*	2	47.05±3.6
rfl2-06-78	*rfl2-99-114*	1	50.6

[a] n, the number of progeny ears analyzed

[b] The mean % of aborted seeds (± standard deviation) per ear, determined by counting all normal (starch-filled) and aborted (empty, collapsed) seeds on each ear; ears contained 284-508 kernels; standard deviations were calculated at http://www.mathportal.org/calculators/statistics-calculator/standard-deviation-calculator.php (accessed 4/12/2017)

Table S4 Recombination distances between chromosome 2L restorer loci

Allele 1	Allele 2	Number of families[a]	% Pollen-sterile progeny[b]	Recombination frequency[c]
Rf3	*rfv*-99-1181*	2	0.8± 0.6	0.016
Rf3	*rfl1-1*	6	9.9±4.8	0.198
Rf3	*rfl2-1*	3	10.7±1.2	0.214
rfl1-1	*rfl2-99-114*	2	1.1±0.2	0.022

[a] Individual families contained 176-241 individuals

[b] Mean % of pollen-sterile progeny (± standard deviation) per family; standard deviations were calculated at http://www.mathportal.org/calculators/statistics-calculator/standard-deviation-calculator.php (accessed 4/12/2017)

[c] Recombination between chromosome 2L restorer alleles estimated by doubling the frequency of pollen-sterile recombinant progeny observed after pollinating CMS-S plants heterozygous for each of two chromosome 2L restorers with an N-cytoplasm non-restoring plant; pollen-sterile recombinants carrying no restorer can be observed phenotypically; pollen-fertile recombinants carrying both restorers in cis cannot be distinguished from the parental genotypes

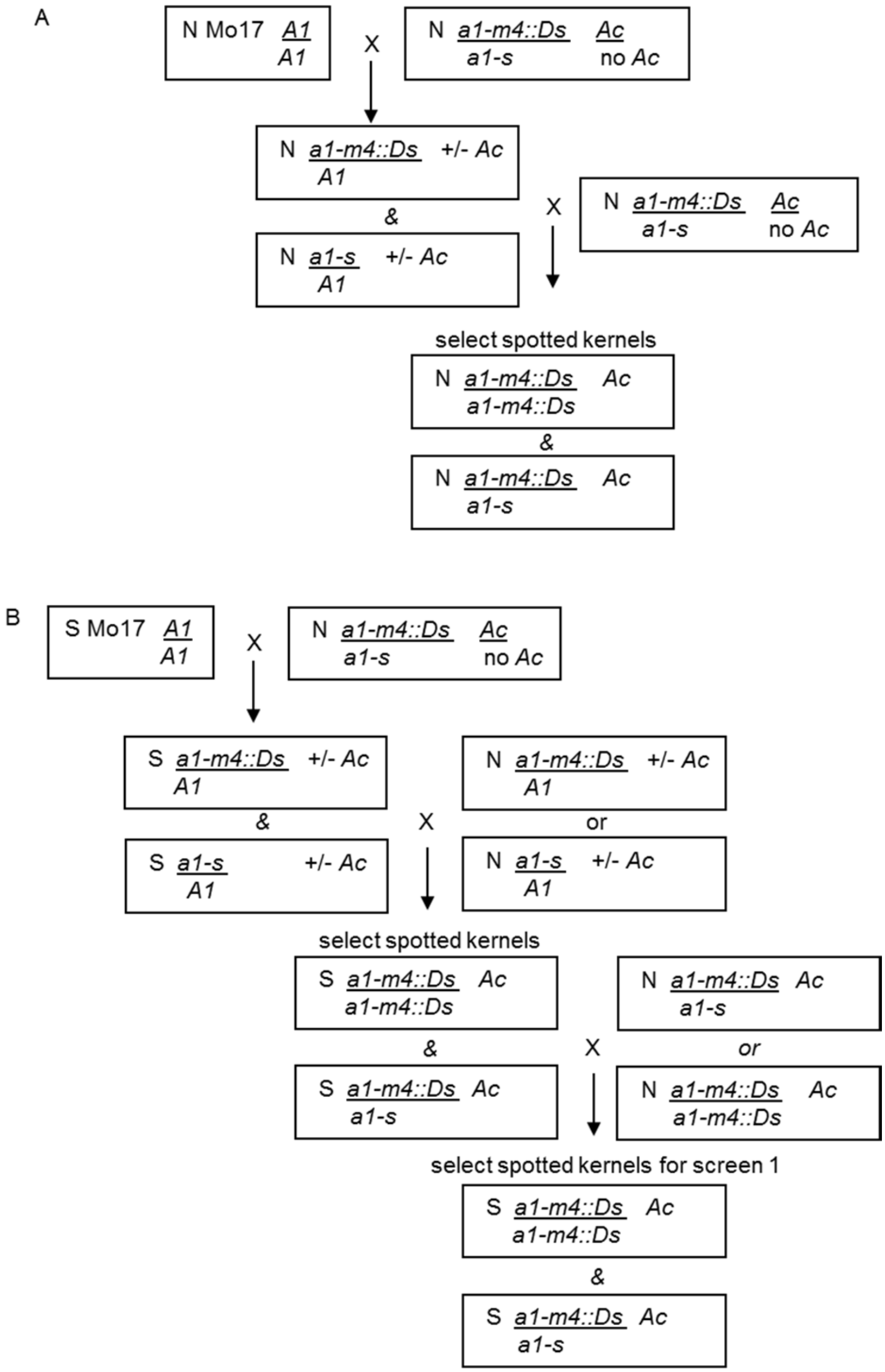

Figure S1 Crosses that generated (A) N-cytoplasm *a1-m4* (*Ac-Ds*) stocks and (B) CMS-S *a1-m4 (Ac-Ds)* populations for restorer mutant screen one.

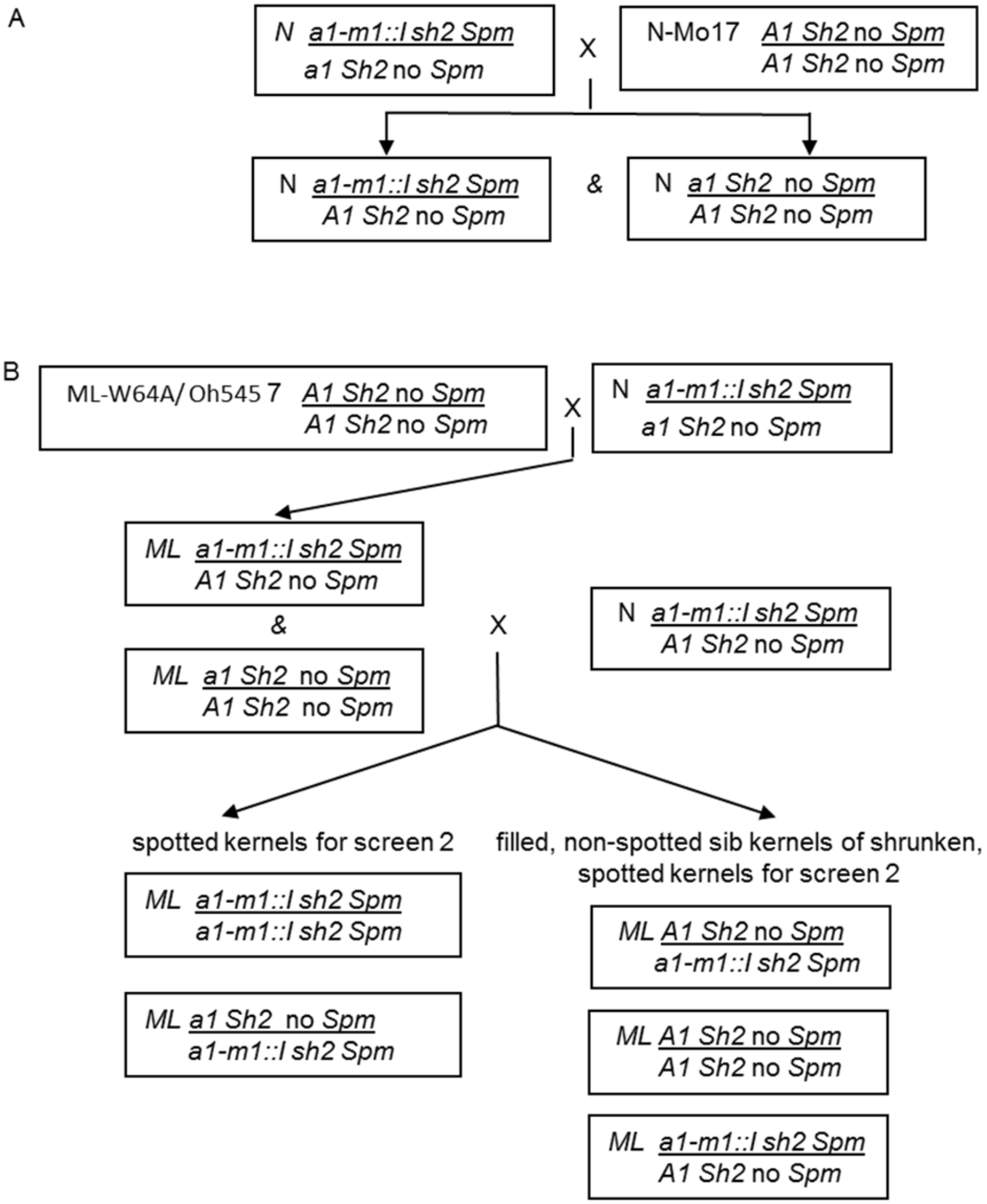

Figure S2 Crosses that generated (A) N-cytoplasm *a1-m14* (*En/Spm*) stocks and (B) CMS-S *a1-m1 (En/Spm)* populations for mutant screen two.

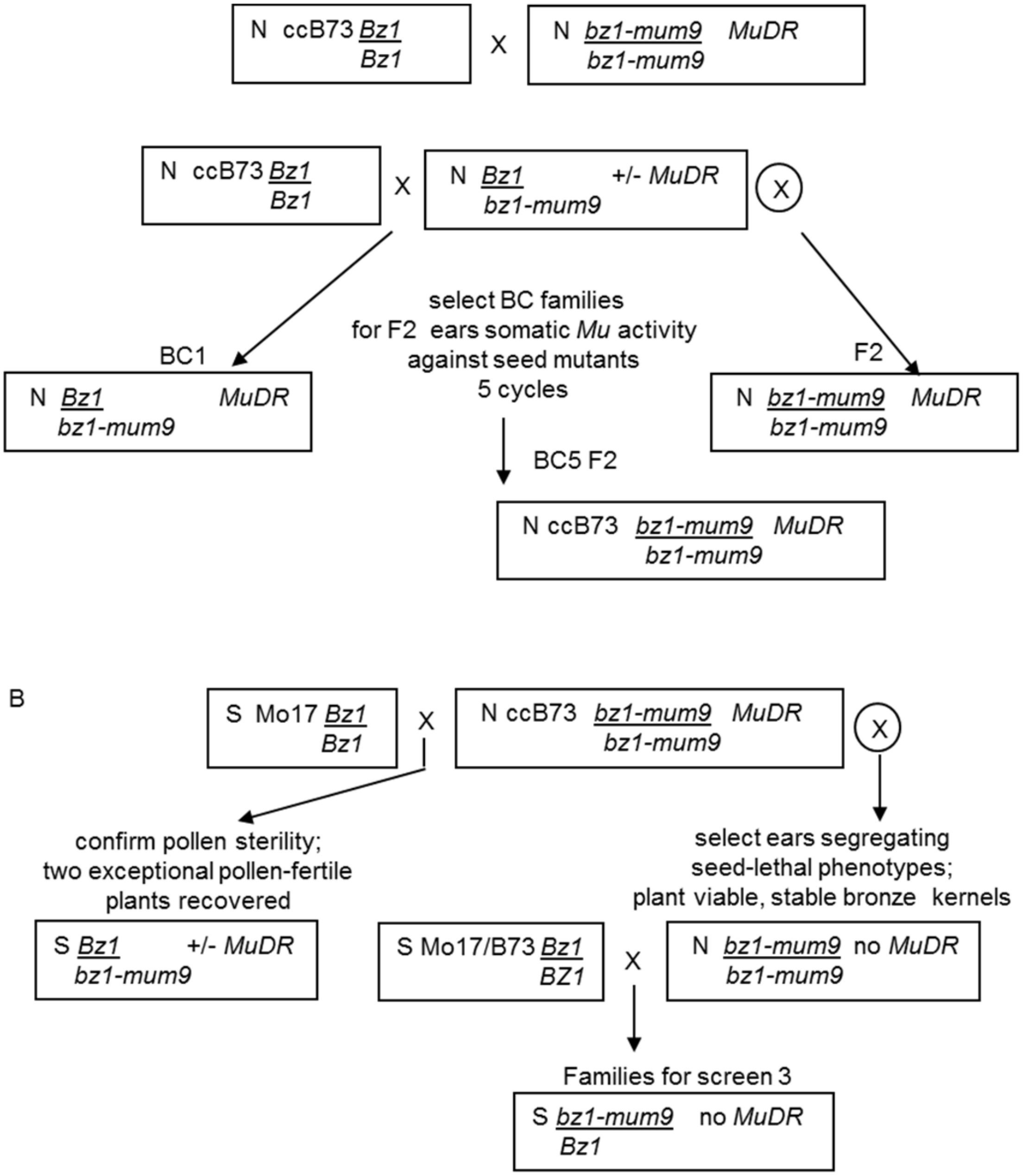

Figure S3 Crosses that generated the N-cytoplasm ccB73 *Mu*-active (*MuDR*) *bz1-mum9* stocks (A). This stock was tested for the absence of endogenous, CMS-S restorers and also used to develop families segregating for seed-lethal phenotypes (B). Viable, stable bronze sibling kernels on ears segregating lethal kernels were then tested for the presence of new restorers for CMS-S in screen three.

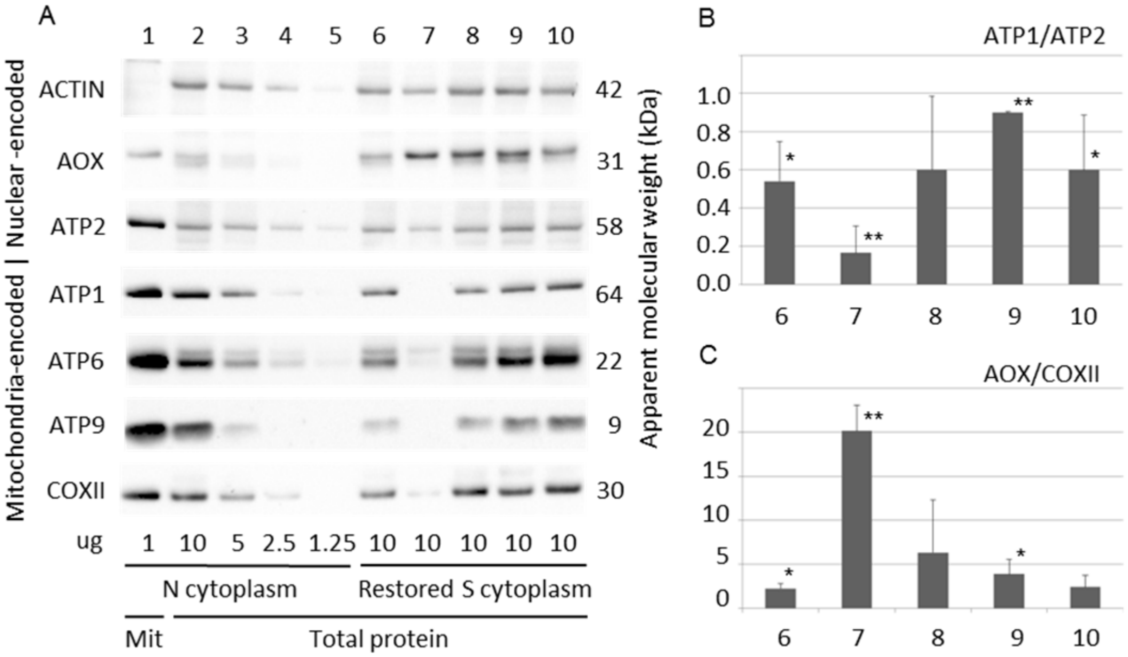

Figure S4 ChemiDoc™ analysis of immunoblots containing proteins extracted from developing CMS-S pollen with homozygous-viable restorer alleles compared to Mo17 N-cytoplasm pollen. (A) ChemiDoc™ images with the proteins detected labeled on the left of each panel and apparent molecular weights indicated on the right. Proteins were immunodecorated and detected following denaturing gel electrophoresis and transfer to nitrocellulose. Sample 1 contained 1 ug of protein extracted from N-cytoplasm Mo17 (Mo17-N) pollen mitochondria. Samples 2-5 contained 10, 5, 2.5 and 1.25 ug of Mo17-N total pollen protein extract, respectively. Samples 6-10 contained 10 ug of total protein extract from S-cytoplasm pollen restored by *Rf3, rfv1-1, rfv*-06-86, rfv*-06-88* and *rfv*-06-89.* Protein samples are biological replicates of those shown in Figure 4. AOX, alternative oxidase; ATP1, ATP2, ATP6 and ATP9, ATP synthase subunits 1, 2, 6 and 9, respectively; COXII, cytochrome oxidase subunit 2. (B) Mean ATP1/ATP2 band volume ratios from two independent samples of CMS-S pollen restored by (6) *Rf3,* (7) *rfv1-1,* (8) *rfv*-06-86,*(9) *rfv*-06-88* and (10) *rfv*-06-89* normalized to the Mo17-N pollen sample ratios. Error bars indicate standard deviations; * and ** indicate ratios different from Mo17-N (=1) at the 0.1 and 0.05 levels, respectively. (C) Mean AOX/COXII band volume ratios from two biological replicate samples of CMS-S pollen restored by (6) *Rf3,* (7) *rfv1-1,* (8) *rfv*-06-86,*(9) *rfv*-06-88* and (10) *rfv*-06-89* normalized to the Mo17-N pollen sample ratios. Error bars indicate standard deviations; * and ** indicate ratios different from Mo17-N (=1) at the 0.1 and 0.05 levels, respectively.

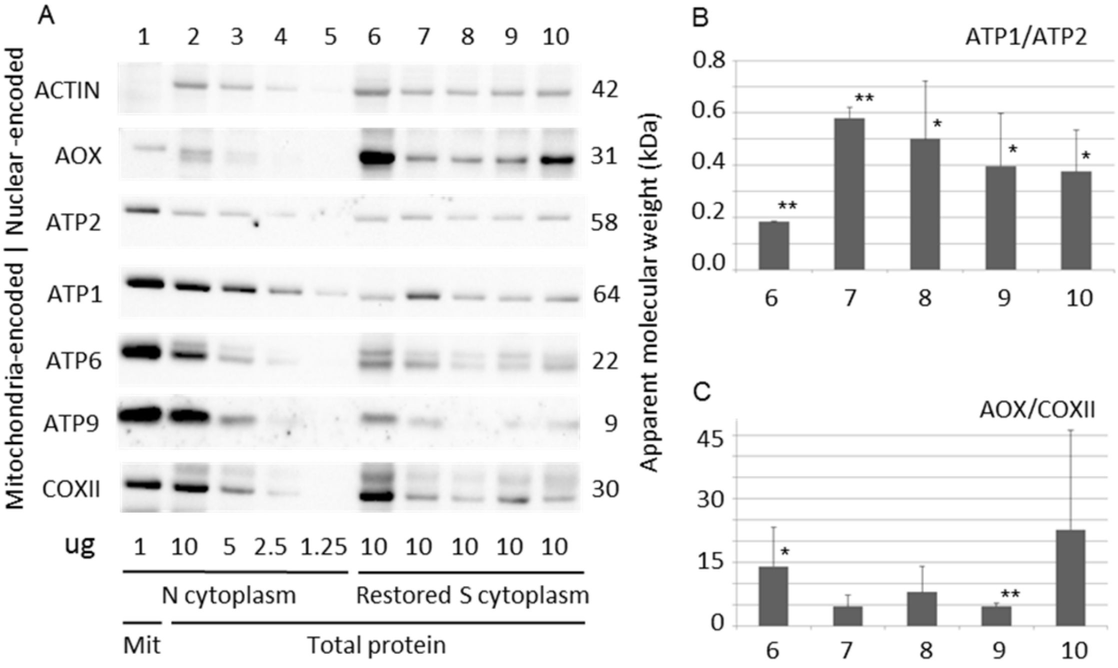

Figure S5 ChemiDoc^{TM} analysis of immunoblots containing proteins extracted from developing CMS-S pollen with *rfl* alleles compared to Mo17 N-cytoplasm pollen. (A) ChemiDoc^{TM} images with the proteins detected labeled on the left of each panel and apparent molecular weights indicated on the right. Proteins were immunodecorated and detected following denaturing gel electrophoresis and transfer to nitrocellulose. Sample 1 contained 1 ug of protein extracted from N-cytoplasm Mo17 (Mo17-N) pollen mitochondria. Samples 2-5 contained 10, 5, 2.5 and 1.25 ug of Mo17-N total pollen protein extract, respectively. Samples 6-10 contained 10 ug of total protein extract from S-cytoplasm pollen restored by *rfl*-06-73*, *rfl*-06-76*, *rfl2-06-78*, *rfl*-06-81*, *and rfl*-06-85*, respectively. Protein samples are biological replicates of those shown in Figure 5. AOX, alternative oxidase; ATP1, ATP2, ATP6 and ATP9, ATP synthase subunits 1, 2, 6 and 9, respectively; COXII, cytochrome oxidase subunit 2. (B) Mean ATP1/ATP2 band volume ratios from two independent samples of CMS-S pollen restored by (6) *rfl*-06-73*, (7) *rfl*-06-76*, (8) *rfl2-06-78*, (9) *rfl*-06-81* and (10) *rfl*-06-85* normalized to the Mo17-N pollen sample ratios. Error bars indicate standard deviations; * and ** indicate ratios different from Mo17-N (=1) at the 0.1 and 0.05 levels, respectively. (C) Mean AOX/COXII band volume ratios from two biological replicate samples of CMS-S pollen restored by (6) *rfl*-06-73*, (7) *rfl*-06-76*, (8) *rfl2-06-78*, (9) *rfl*-06-81* and (10) *rfl*-06-85* normalized to the Mo17-N pollen sample ratios. Error bars indicate standard deviations; * and ** indicate ratios different from Mo17-N (=1) at the 0.1 and 0.05 levels, respectively.
